# Deep Active Learning Framework for Lymph Node Metastasis Prediction in Medical Support System

**DOI:** 10.1155/2022/4601696

**Published:** 2022-05-10

**Authors:** Qinghe Zhuang, Zhehao Dai, Jia Wu

**Affiliations:** ^1^School of Computer Science, Central South University, Changsha 410083, China; ^2^Department of Spine Surgery, The Second Xiangya Hospital, Central South University, Changsha 410011, China; ^3^Research Center for Artificial Intelligence, Monash University, Melbourne, Australia

## Abstract

Assessing the extent of cancer spread by histopathological analysis of sentinel axillary lymph nodes is an important part of breast cancer staging. With the maturity and prevalence of deep learning technology, building auxiliary medical systems can help to relieve the burden of pathologists and increase the diagnostic precision and accuracy during this process. However, such histopathological images have complex patterns that are difficult for ordinary people to understand and require professional medical practitioners to annotate. This increases the cost of constructing such medical systems. To reduce the cost of annotating and improve the performance of the model as much as possible, in other words, using as few labeled samples as possible to obtain a greater performance improvement, we propose a deep learning framework with a three-stage query strategy and novel model update strategy. The framework first trains an auto-encoder with all the samples to obtain a global representation in a low-dimensional space. In the query stage, the unlabeled samples are first selected according to uncertainty, and then, coreset-based methods are employed to reduce sample redundancy. Finally, distribution differences between labeled samples and unlabeled samples are evaluated and samples that can quickly eliminate the distribution differences are selected. This method achieves faster iterative efficiency than the uncertainty strategies, representative strategies, or hybrid strategies on the lymph node slice dataset and other commonly used datasets. It reaches the performance of training with all data, but only uses 50% of the labeled. During the model update process, we randomly freeze some weights and only train the task model on new labeled samples with a smaller learning rate. Compared with fine-tuning task model on new samples, large-scale performance degradation is avoided. Compared with the retraining strategy or the replay strategy, it reduces the training cost of updating the task model by 79.87% and 90.07%, respectively.

## 1. Introduction

Accurate breast cancer staging is an essential task performed by pathologists worldwide to inform clinical management [1]. The histopathological analysis is the gold standard for precancerous lesion diagnosis. It has very high accuracy and reliability for diagnosis. Assessing the extent of cancer spread by the histopathological analysis of sentinel axillary lymph nodes is an important part of breast cancer staging. However, this assessment process is tedious, time-consuming, and prone to make mistakes when handled by pathologists. With the development of artificial intelligence technologies and the prevalence of auxiliary medical diagnostic systems based on them [2–8], developing an auxiliary system for the detection of lymph node metastases in breast cancer is feasible and valuable. It could result in a significant reduction in the workload of pathologists.

**Figure 1:**
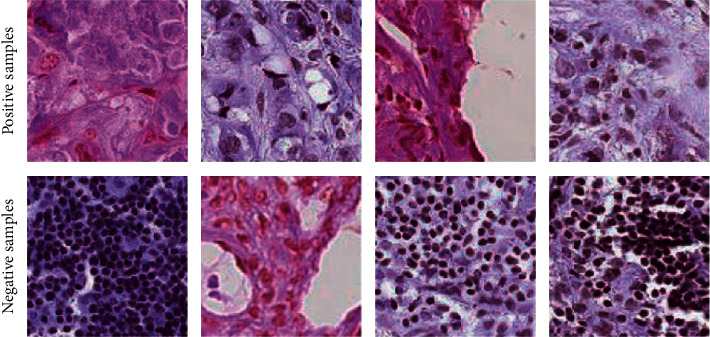
Histopathological scans of lymph nodes. These patterns are complicated and hard for nonprofessional people to distinguish.

The construction of such systems generally relies on supervised learning technology. However, supervised learning requires a large number of labeled samples. Histopathological scans of lymph nodes are complex, as shown in Figure 1. It is not easy to find deterministic features in human eyes. Therefore, nonprofessional people can hardly distinguish between positive and negative types. This complexity makes the construction of such diagnostic systems or other sophisticated medical systems require a large number of labeled samples to train on the one hand and consumes a lot of resources, especially precious medical resources for annotating. When resources are limited, building such a medical system is very challenging.

Fortunately, although there is a lack of labeled samples and the cost of labeling is high, there are currently a large number of unlabeled samples in hospitals, and some useful information can be obtained from these unlabeled samples. To alleviate the limitation of insufficient labeled data, researchers have proposed different kinds of methods including active learning. Active learning [9] is an effective method to solve the problem of lacking labeled data. It is an iterative process that follows three steps. First, the model is trained with a labeled small dataset. Second, the most informative samples are selected from unlabeled data based on some strategy and sent to human experts for labeling. Then, the model is retrained by the new training data. Samples selected based on specific strategies aim to quickly improve the performance of the original model. The application scenarios are suitable for auxiliary medical support systems: insufficient initial training set (system builders do not have enough labeled data at first and have to collect data or annotate data before system construction), an enormous quantity of unlabeled data (amount of preserved data in hospital databases is huge but have few labels), and expensive annotation cost (medical image usually needs annotation from professional practitioners). Therefore, active learning is applied widely in medical informatic fields [10–12].

At present, most strategies are based on the uncertainty of the model to the sample, such as least confidence, margin sampling, and entropy [13]. Compared with blindly spending time and energy on labeling data, active learning can improve model performance with a smaller labeling cost [14].

However, most active learning frameworks have two defaults that limit their application. The first one is that the selection strategies are not efficient enough. This is provoked by the similar samples selected in one batch and will decrease the annotating efficiency. To overcome this shortcoming, we proposed a hybrid three-stage selection, which aims to reduce the sample redundancy caused by the uncertainty selection method. Besides, this hybrid strategy selects samples that can eliminate the distribution difference between labeled data and unlabeled data quickly and improves the annotation efficiency further.

The other default is that most active learning frameworks rely on retraining to update the task model. This is because it is difficult for neural networks to acquire incremental knowledge. Training new tasks or new data on the old neural network will lead to a sharp drop in performance on the original data or tasks. This phenomenon is called catastrophic forgetting [15]. It is very serious, especially when the task types or data domains have great differences. While in the active learning iteration process, the data distribution difference between the newly labeled samples and the old labeled samples may be small, it will also lead to performance degradation, which is called concept shift [14]. Retraining is a simple way to avoid concept shift but has high time and computation costs, which will cause obstacles in some application scenarios. In this study, we investigate a new method that reduces the performance drop and training cost simultaneously.

The main innovation or contribution of the study includes the following:

- We constructed a classification system for breast cancer lymph node metastasis prediction based on deep active learning and proposed a new three-stage selection strategy. Different from the traditional uncertainty-based strategy, a diversity strategy is introduced to reduce data redundancy. Meanwhile, distribution differences between labeled and unlabeled samples are measured to reduce the distribution difference. This hybrid strategy obtains higher annotating efficiency compared with uncertainty-based strategies or diversity-based strategies.
- We explore a new incremental approach for model updating. Different from the general active learning iteration process that uses all the labeled data to retrain the model, we use a freezing and fine-tuning method to ensure that the model acquires new knowledge, while reducing the forgetting of the original knowledge. We believe that this new update method will expand application scenarios of active learning, especially under the tendency of a larger model and enormous data.

The rest of this study is organized as follows: Section 2 summarizes the research work in related fields, Section 3 introduces the method used in this study, Section 4 is the experiment and result analysis, and Section 5 is the conclusion.

## 2. Related Works

Active learning has been widely combined with deep learning models due to its significant reduction in labeling costs [16–19]. Yang et al. [10]combined active learning with a fully convolutional neural network for segmentation tasks on lymph node ultrasound images and finally achieved and trained using only 50% of the labeled samples. Smailagic et al. [17] used active learning and convolutional neural networks to classify fundus blood vessel images, melanoma images, and breast cancer pathology images. The experimental results showed that the model combined with active learning strategy can only use 25% of the labeled data to train the model. It still achieves an accuracy rate of 6.3% higher than the base model under the same conditions. Zhao et al. [18] used an active learning framework based on the U-Net model to segment hand bone images and only used 43.16% of the labeled samples to achieve the same effect as training with all the labeled samples. Zhou et al. [19] used active learning for colonoscopy frame classification, polyp detection, and pulmonary embolism detection, reducing the labeling cost by 82%, 86%, and 80%, respectively. These applications fully demonstrate the effectiveness of active learning.

A typical active learning process [20, 21] is composed of a dataset, a model, and experts or oracles for the model to query. The dataset in active learning is generally made up of a small number of labeled samples and a large number of unlabeled samples. The model is first trained on the labeled dataset, and then, based on a certain strategy some samples are selected from the unlabeled data and given to experts for labeling. The new labeled data are put into the training set for retraining the model. This process iterates until a certain convergence condition, such as the performance meets the requirements, or the labeling cost exceeds budget.

The core of active learning is to design a selection strategy so that the labeled samples can effectively improve the model performance. The classic selection strategy is based on model uncertainty [22, 23].

Many researchers have carried out research based on uncertainty. For example, Wang et al. integrated active learning with the training process of deep belief networks for the first time, introduced a loss function specific to active learning tasks, and trained the model to minimize the loss function. Houlsby et al. [24] proposed the Bayesian active learning by disagreement (BALD) uncertainty, which is mainly used in the Bayesian networks. Gal et al. [25] proposed the MC-dropout method as a proxy for BALD, which obtains model perturbations by turning on dropout during prediction so that BALD uncertainty can be captured in general convolutional networks. Gal et al. [26] validated the effectiveness of the MC-dropout method on high-dimensional image data. William et al. [22] used an ensemble-based method to measure the uncertainty of convolutional neural networks, which integrates the results of multiple convolutional neural networks to obtain the uncertainty measure of the model, which is better than the geometry-based method, and faster performance improvement based on the MC-dropout [25] method. Zhao et al. [18] used the output difference in the middle layers of the network to measure the uncertainty of the convolutional neural network on the segmentation task. In particular, the Dice index is calculated from the output of the previous layer in the network, the output of the middle layer of the network, and the output of the final layer, and the average of the two is taken as the uncertainty proxy. Experiments show that the proxy uncertainty and the true Dice index exhibit a significant correlation, which can be used as an uncertainty measure; that is, the larger the calculated average Dice index, the smaller the uncertainty.

However, the use of uncertainty-based strategies in neural networks is generally to select a batch of samples at a time. The uncertainty-based strategies cannot deal with sample redundancy and often select a batch of samples that contains many similar samples, which reduces the labeling efficiency. Therefore, strategies based on representation or diversity are proposed.

**Figure 2:**
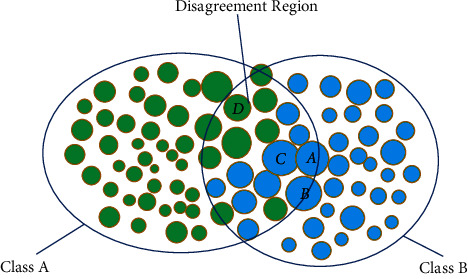
Illustration of active learning selection strategy.

The representative strategy aims to pick representative samples for annotation so that the model has a better understanding of the overall data distribution. As shown in Figure 2, the green circles represent class A, and the blue circles represent class B. The size of the circle represents the uncertainty of the sample model. Generally speaking, the decision boundary of disagreement regions (intersection regions) is complex, so annotating samples in disagreement regions will obtain higher performance improvement. The samples selected by the uncertainty-based strategy may be clustered together; for example, three samples A, B, and C may be selected based on uncertainty, while A and *D* may be selected by the representative-based strategy. Samples A and *D* are more useful for the model to understand overall data distribution, so they tend to achieve higher performance improvement. There are many active learning application cases based on representational strategies [27, 28].

Rather than using the representative strategy alone, a hybrid strategy combining representative and uncertainty strategies is used more often [29–34]. Yang et al. [16] trained a cluster of models by replacing the labeled data, using the output variance of each model to measure the uncertainty, and using the intermediate output layer of the convolutional neural network as the representation of the image. The similarity of the representation was used as a metric of similarity between images. Then, a greedy strategy is used to select batches with a small similarity between samples for annotating. Andreas et al. [31] proposed BatchBALD. Different from the general BALD selection strategy, which is only based on the BALD score, BatchBALD selects samples one by one and calculates the mutual information between the selected samples every time. Among the unlabeled samples, the mutual information between the selected sample and the currently to-be-labeled sample is the smallest, so that the sample diversity in a selection batch constructed greedily is the largest, but there is no guarantee that the selected batch is the most diverse among all possible combinations. Fedor et al. [29] also combined uncertainty and diversity. First, a batch of samples with large uncertainty was selected, and then, the samples with large uncertainty were clustered to select samples that are nearest to the class center. Experiments on text and image datasets show that it outperforms strategies using uncertainty strategies and clustering alone. Jordan et al. [33] proposed an adaptive gradient embedding method, which uses the gradient size of the last layer of the model to represent uncertainty and takes into account uncertainty and diversity by embedding samples into the gradient space and performing clustering. The benefit of this approach is that clustering based on the gradient space automatically balances uncertainty and diversity without manual tuning of other hyperparameters and thus has better adaptability to different batch sizes. Zhou et al. [19] used the difference between the output of the rotation-augmented image and the original image of the classifier to measure the uncertainty and used the class difference in the samples within the batch as the diversity measure. Sampling probability is explicitly calculated before sampling from unlabeled data.

## 3. Methodology

**Figure 3:**
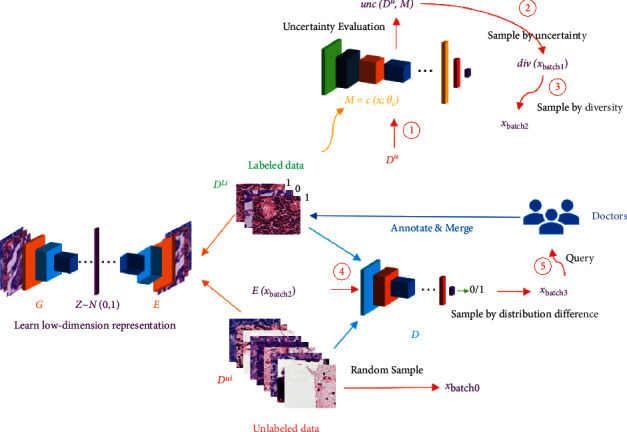
Detailed structure of the proposed deep active learning framework.

Figure 3 shows the general process of our proposed framework. We use the proposed three-stage selection strategy, aiming to obtain samples with large uncertainty, low redundancy, and can quickly eliminate the distribution difference between labeled samples and unlabeled samples. Each stage focuses on a selection indicator, namely uncertainty, sample diversity, and distribution difference between labeled samples and unlabeled samples. Overall, the selection strategy is still an improvement based on uncertainty. Traditional uncertainty-based strategies face the problem of high sample redundancy. As described in Section 2, many works incorporate diversity strategies and balance the weights of the two explicitly or implicitly. On this basis, we added a selection criterion for the distribution difference between labeled samples and unlabeled samples. The motivation of this selection criterion is that due to the model's preference for data, the distribution difference between labeled samples and unlabeled samples will become larger and larger, and reducing this distribution difference will help speed up performance improvement.  Section 3.1 describes each component in Figure 3 in detail and the overall workflow.  Section 3.2 describes the specific implementation process in each stage.

### 3.1. Components and Workflow

#### 3.1.1. Task Model

Breast cancer lymph node prediction is a classification problem, and we use convolutional neural networks as a classification model. The breast cancer lymph node image and its category are represented by *x* and *y*, respectively, the classification network is represented by ℳ, the parameter is *θ*_ℳ_, and the predicted class $\hat{y}=ℳ\left(x\right)$. ℳ is optimized according to the following equation:

(1) $$\begin{array}{c} \arg\min_{\theta_{ℳ}}l_{ℳ}\left(\hat{y},y\right). \end{array}$$

#### 3.1.2. Labeled and Unlabeled Datasets

The labeled sample is defined as *𝒟*^ℒ^, the unlabeled sample is defined as *𝒟*^*𝒰*^, and then the total sample is *𝒟*= *𝒟*^ℒ^∪*𝒟*^*𝒰*^. The initial labeled sample is marked as *𝒟*^ℒ_0_^, the labeled sample in the *i*th round is *𝒟*^ℒ_*i*_^, and the unlabeled sample is *𝒟*^*𝒰*_i_^. The goal of active learning is to design a selection strategy 𝒬, using 𝒬 selects out *𝒟*^*𝒰*_I_^ from *𝒟*^𝒬_*i*_^, where *𝒟*^𝒬_*i*_^ is the sample selected and sent to the expert for annotation in the *i*th iteration. After *𝒟*^ℒ_*i*_^=*𝒟*^ℒ_*i*−1_^∪*𝒟*^𝒬_*i*_^ can change to *𝒟*^ℒ_*i*_^. The selection strategy 𝒬 follows the following equation:

(2) $$\begin{array}{c} {\text{argmin}}_{D^{Q_{i}}\subseteq D^{U_{\text{i}}},\left(x,y\right)\in D^{Q_{i}}}E_{\left(x,y\right)}l_{ℳ}\left(ℳ\left(x\right),y\right), \end{array}$$

where *l*_ℳ_(·) is the loss function of task model ℳ.

#### 3.1.3. Auto-Encoder

In addition, we need to learn a representation of the global distribution of samples. Embedding the samples into a low-dimensional space is conducive to measuring the representative of the samples. At the same time, it is helpful to distinguish whether *𝒟*^ℒ^ and *𝒟*^*𝒰*^ are from the same distribution. We use an auto-encoder to complete this operation. A well-learned auto-encoder is beneficial to improve the accuracy of diversity metrics and reduce the learning difficulty of the distribution discriminator. The auto-encoder is divided into two parts: the encoder and the decoder, which are represented by *E* and *G*, respectively, and its network parameters are represented by *θ*_*G*_ and *θ*_*E*_, respectively. *E* is responsible for encoding, *z*=*E*(*x* ), and *G* is responsible for reconstructing the original image using the encoding result of *E* or *z*. We expect the size of *z* to be smaller than the size of the original *x*. The optimization of *θ*_*G*_ and *θ*_*E*_ follows the following expression:

(3) $$\begin{array}{c} {\text{argmin}}_{\theta_{G},{\;\theta }_{E}}E_{x\in \;D^{ℒ}\cup D^{U}\;}l_{AE}\left(G\left(E\left(x\right)\right),x\right), \end{array}$$

where *l*_*AE*_(·) is the loss function of the auto-encoder, generally mean square error. In (3), the auto-encoder uses all the data (*𝒟*^ℒ^∪*𝒟*^*𝒰*^) for training without adding additional loss terms other than the reconstruction loss. The reason for emphasizing this is that this ensures that the auto-encoder treats the labeled samples and unlabeled samples fairly, and there is no bias. So, we can think that the learned low-dimensional variable *z* is subject to the same distribution on *𝒟*^ℒ^ and *𝒟*^*𝒰*^, although *z* does not necessarily obey *𝒩*(0,1) (in VAE [35], *z* is bound to a fixed distribution to facilitate sampling from *z* to obtain fake data, and we do not need to obtain fake data, so we can focus on to optimize the reconstruction loss, regardless of the distribution of the latent variable *z*).

#### 3.1.4. Discriminator

The discriminator *D* is used to measure the distribution difference between *𝒟*^*𝒰*^ and *𝒟*^ℒ^ during each iteration, it receives *z* as input, and the output sample belongs to *𝒟*^*𝒰*^ or *𝒟*^ℒ^. This is a self-supervised process without labeling. The discriminator follows a general classification neural network.

#### 3.1.5. Doctors (Oracle)

After completing the data selection, professional personnel is needed for annotation. In the breast cancer lymph node classification problem, this role is generally doctors. By annotating new samples, they help the model acquire new knowledge and improve performance. The biggest advantage of active learning is to reduce the number of annotations in situation that needs professional but expensive annotation, thereby reducing the cost of building task models. In the experiment section, annotation by doctors is simulated by database queries.

### 3.2. Proposed Query Strategy

The query strategy is the core of active learning. We have designed a three-stage active learning selection strategy. The entire selection process is marked with red arrows in Figure 3 and is divided into 5 steps, which are marked with ➀-➄, respectively. In the  *i*th iteration, we first use *𝒟*^ℒ^ to train task model ℳ and then calculate the uncertainty of *𝒟*^*𝒰*^ according to ℳ, denoted as *unc*(*𝒟*^*u*^, ℳ) (Step 1). *unc*(·) is the uncertainty metric.

Samples with large uncertainty were selected from *𝒟*^*𝒰*^ and are recorded as *x*_*batch*1_ (Step 2) where samples have high uncertainty, but maybe similar, as described in Section 2. Next, the representative of *x*_*batch*1_ is evaluated, and the most representative samples are selected and recorded as *x*_*batch*2_ (Step 3), which is a subset of *x*_*batch*1_. Then, we encode *x*_*batch*2_ with the pretrained encoder *E* to obtain *E*(*x*_*batch*2_), and discriminator *D* is used to evaluate distribution difference and obtain *D*(*E*(*x*_*batch*2_)). *x*_*batch*3_ is obtained by sorting *D*(*E*(*x*_*batch*2_)). *x*_*batch*3_ is the final selected *𝒟*^𝒬_*i*_^. After querying its label (Step 5), it is then merged with the existing labeled dataset *𝒟*^ℒ_*i*_^. The entire query process is completed.

#### 3.2.1. Uncertainty

The first stage of the selection strategy is selected based on uncertainty.

The uncertainty-based query strategy is the most basic and most commonly used. Deep active learning is an active learning method based on deep learning models, which involves a measure of uncertainty in neural networks. Generally, a very natural idea is to regard the output of the neural network as a probability distribution, from which a variety of uncertainty measurement methods are derived, such as least confidence, entropy, margin sampling, and BALD method.

Assume that the probability of sample *i* belongs to category *c* is ${\hat{p}}_{c}$, and *𝒞* is the set of all categories. Then, for least confidence, the uncertainty is measured according to the following equation:

(4) $$\begin{array}{c} {\text{unc}}_{LF}\left(x_{i},\;ℳ\right)={\text{min}}_{c\in C}\left({\hat{p}}_{c}\right). \end{array}$$

However, neural networks tend to be overconfident in their prediction results. Therefore confidence-based methods are not good.

Entropy-based uncertainty is calculated by the entropy of the output probability distribution of the neural network as follows:

(5) $$\begin{array}{c} {\text{unc}}_{\text{entropy}}\left(x_{i},ℳ\right)=\sum_{c}{\hat{p}}_{c}\text{log}\left({\hat{p}}_{c}\right). \end{array}$$

Margin sampling uncertainty is calculated by the probability difference between the class with the largest confidence and the class with the next largest confidence as follows:

(6) $$\begin{array}{c} {\text{unc}}_{\text{MS}}\left(x_{i},ℳ\right)={\hat{p}}_{c_{1}}-{\hat{p}}_{c_{2}}, \end{array}$$

where $c_{1}={\text{argmin}}_{c\in 𝒞}\;{\hat{p}}_{c}$ and $c_{2}={\text{argmin}}_{c\in \;𝒞\backslash c_{1}}\;{\hat{p}}_{c}$.

BALD uncertainty is measured by opening the dropout layer during the prediction process and performing multiple dropouts as follows:

(7) $$\begin{array}{c} {\text{unc}}_{\text{BALD}}\left(x_{i},ℳ\right)=-\sum_{c}\left(\frac{1}{T}\sum_{t}{\hat{p}}_{c}^{t}\right)log\left(\frac{1}{T}\sum_{t}{\hat{p}}_{c}^{t}\right)+\frac{1}{T}\sum_{c,t}{\hat{p}}_{c}^{t}\log\left({\hat{p}}_{c}^{t}\right), \end{array}$$

where *T* is the total number of predictions and ${\hat{p}}_{c}^{t}$ is the probability that sample *i* belongs to  *c* in *t*th predictions. Since multiple predictions are required, it often takes a long time expense.

In this study, we use uncertainty based on margin sampling.

#### 3.2.2. Diversity

The second stage is to select based on sample representative or diversity. This approach is inspired by the fact that uncertainty strategies focus on uncertainty and select many similar samples. Performing secondary selection based on sample representative will help to improve the selection efficiency. We model the selection of representative samples as the k-center problem. The k-center problem aims to select *k* centers from a dataset to minimize the maximum distance from other points to the nearest center point. The whole dataset can be represented by *k*-center points. Here our purpose is to reduce the redundancy of samples in *x*_*batch*1_, so that *x*_*batch*2_ and *𝒟*^ℒ_*i*_^ can represent *x*_*batch*1_, and this process can be described as follows:

(8) $$\begin{array}{c} \left(x_{\text{batch}2},\delta \;\right)={\text{min}}_{D^{{ℒ}_{i}}\cup x_{{\text{batch}}_{2}}}{\text{max}}_{x_{i}\in x_{{\text{batch}}_{1}}}{\text{min}}_{x_{j}\in D^{{ℒ}_{i}}\cup x_{{\text{batch}}_{2}}}\text{dis}\left(x_{i},x_{j}\right), \end{array}$$

where dis(·) is distance metric and *δ* is the minimum distance between center points and non-center points. Here, it is based on the L2 distance of the embedding of previously trained auto-encoder, namely:

(9) $$\begin{array}{c} \text{dis}\left(x_{i},x_{j}\right)={\left|\left|E\left(x_{i}\right)-E\left(x_{j}\right)\right|\right|}_{2}. \end{array}$$

**Figure 4:**
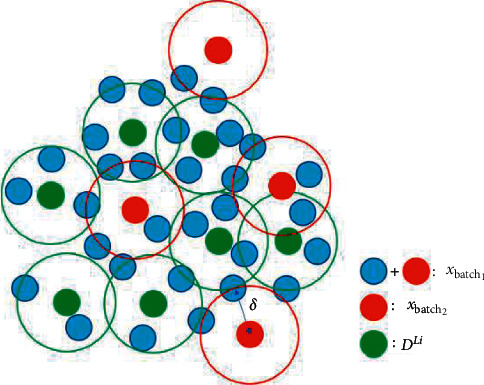
Schematic diagram of sample selection based on k-center.

This process is depicted in more detail in Figure 4. Each circle represents a sample point. Points surrounded by a larger circle with a radius of *δ* are the center points. The green point represents *𝒟*^ℒ_*i*_^. The red and blue points together form *x*_batch_1__. Red and green points are the center points of all sample points. The red point is the result *x*_batch_2__.

However, the k-center problem is NP-hard. In practice, we use the improved greedy algorithm proposed by [26]. We can formulate this process as follows:

(10) $$\begin{array}{c} x_{{\text{batch}}_{2}}=k\_\text{center}\left(x_{{\text{batch}}_{1}},D^{{ℒ}_{i}}\right). \end{array}$$

#### 3.2.3. Distribution Difference

The initial labeled samples *𝒟*^ℒ_0_^ and unlabeled samples *𝒟*^*𝒰*_0_^ are randomly sampled from *𝒟*, so there is no distribution difference between *𝒟*^ℒ_0_^ and *𝒟*^*𝒰*_0_^, but with the biased selection of *𝒟*^*𝒰*_*i*_^ based on ℳ, there will be a distribution difference between *𝒟*^ℒ_*i*_^ and *𝒟*^*𝒰*_*i*_^.

In the third stage, our goal is to use a small number of labeled samples to represent unlabeled samples, so *𝒟*^ℒ_*i*_^ and *𝒟*^*𝒰*_*i*_^ need to obey the same distribution. The purpose of the third stage of selection is to select samples from *𝒟*^*𝒰*_*i*_^ that have the most dissimilar distribution with *𝒟*^ℒ_*i*_^.

We do not need to know what distribution *𝒟*^ℒ_*i*_^ and *𝒟*^*𝒰*_*i*_^ follow, and we just need to determine whether they are the same. This can be obtained by training a discriminator whose functions are similar to the discriminator in GAN [36]. In GAN, a discriminator is used to discriminate whether a sample is real or synthetic. Here, it is used to determine a sample from *𝒟*^ℒ_*i*_^ or *𝒟*^*𝒰*_*i*_^. We input the results of the encoder into the discriminator *D* for training, and the training loss is as follows:

(11) $$\begin{array}{c} L_{D}=-\left(log\left(D\left(E\left(D^{{ℒ}_{i}}\right)\right)\right)+log\left(1-D\left(E\left(D^{U_{\text{i}}}\right)\right)\right)\right). \end{array}$$

This will force *D* to output 0 for *E*(*𝒟*^*𝒰*_I_^) and 1 for *E*(*𝒟*^ℒ_*i*_^).

When querying, *E*(*x*_*batch*_2__) is input into *D* and the point is picked with the smallest output value. The final obtained *x*_*batch*_3__ is *𝒟*^𝒬_*i*_^. *𝒟*^𝒬_*i*_^ is sent to experts for annotation and combined with *𝒟*^ℒ_*i*_^ as *𝒟*^ℒ_*i*+1_^, while removing *𝒟*^𝒬_*i*_^ from *𝒟*^*𝒰*_I_^ to form *𝒟*^*𝒰*_*i*+1_^.

In summary, the entire process can be summarized as Algorithm 1.

### 3.3. Update Strategy

There are two ways to update the model, one is retraining: reinitializing the model, using all the labeled data for training, and the other is to update incrementally, using part of the labeled data to update the original task model.

Retraining gives the newly added samples the same weight as the original samples so that the model is neither hindered by the deviations learned from the old samples, nor affected too much by the new samples. The overall data distribution is more accurately grasped, and therefore, it is widely used. However, the time cost of retraining is huge. As the iteration process increases, the size of the labeled dataset also increases, and the cost of retraining each time is high.

Therefore, we use a fine-tuning-based method to update the model. It is different from the general fine-tuning method. It not only reduces the learning rate but also adds some dropout layers. During the first training, these dropout layers preserve all the weights. When the model is updated, only the newly labeled data are sent for training. Meanwhile, dropout layers are turned on to suppress some neurons with a certain probability.

## 4. Experiments

### 4.1. Implementation Details

We define Conv(*x*, *y*) to denote a convolutional layer, which consists of a 2D convolutional operation with *x* kernels each having a *y* × *y*  size, a batch norm operation, an activation operation by ReLU function, and a 2 × 2 max pool operation with the kernel of *x* × *y*; *FC*(*x*) to denote a fully connected layer, which has *x*  output units activated by ReLU function; and DP(*p*) as dropout layer with the probability of *p* to reserve the units.

The task model for the PCam dataset can be formulated as Conv(32,3), Conv(64,  2), Conv(128,  3), Conv(256,  3), Conv(512,3), DP(0.5), FC(1024), DP(0.5), FC(512), DP(0.4), and FC(2). The encoder part for the auto-encoder of the PCam dataset is acquired by deleting the last four layers based on the task model, and the decoder part is the reversed version (the convolution is replaced with transposed convolution and the structure is inverted) of the encoder. The structure of task models for MNIST and CIFAR10 are as follows [33], and auto-encoders are built in a similar procedure to PCam. Suppose the embedding dimension of the encoder is *d*, the discriminators' structure follows FC(2*d*), FC(3*d*), FC(2*d*), DP(0.5), FC(*d*), DP(0.5), and FC(1).

All the datasets are split into training set, validation set, and testing set. We randomly preserve 7,000 samples and 3, 000 samples for testing and evaluation. After each epoch of training, the task model is evaluated and saved. The final testing performance is calculated on the model with the best evaluation performance. Each experiment is carried out 3 times with different dataset splits. We use the Adam optimizer with a learning rate of 0.0001. The training process is stopped if the evaluation performance does not increase in 20 epochs.

When updating by the proposed method, we add extra DP(*p*) layer after layers not followed by dropout layers and set *p*=1 for training and *p*=0.7 for fine-tuning. We fine-tune 20 epochs in the proposed method and fine-tuning method.

### 4.2. Effectivity of Proposed Strategy

First, we conducted experiments to prove the effectiveness of the proposed framework on the public PatchCamelyon dataset [37] (PCam). The PatchCamelyon dataset consists of 327,680 color images (96 × 96px) extracted from histopathological scans of lymph node sections. Each image is annotated with a binary label indicating the presence of metastatic tissue.

The PCam dataset has a large amount of data. It is difficult to find such a large dataset in the real application. Therefore, we only use 50,000 training data as the total number of training samples, of which positive and negative samples account for the same proportion.

We compare the proposed three-stage hybrid strategy with uncertainty-based strategies, including entropy (5), confidence (4), margin sampling (6), and representative-based strategy coreset [27].

**Figure 5:**
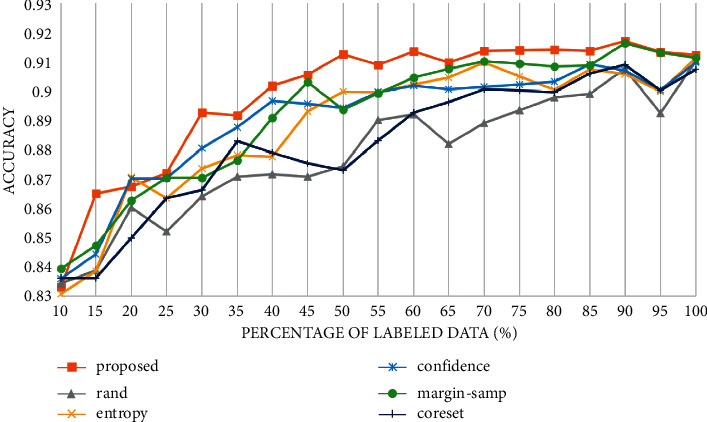
Accuracy curve of different selection strategies on PCam.

In the experiment, 10% of the total training samples are selected as the initial training set, and then, 5% of the samples are annotated according to a specific query strategy in each iteration. The accuracy curve is recorded as shown in Figure 5. All strategies use the same structure of the classification model. When querying by the proposed query strategy, 15%, 10%, and 5% of the total samples are selected at each stage respectively. If the remaining samples are less than 15% or 10%, all the remaining samples are selected.

As shown in Figure 5, both the uncertainty-based strategy and the representation-based strategy are better than random selection. In the first iteration, our strategy achieves much higher accuracy than other strategies. In the entire iterative process, our strategy can improve the accuracy by up to 3.8% (when the labeled dataset accounts for 50%) compared with the random selection strategy and at 1.2% (when the labeled dataset accounts for 30%) compared with other selection strategies. When the labeled dataset reaches 50%, the accuracy achieved by our strategy already exceeds the accuracy trained with the entire dataset, while the uncertainty-based strategy outperforms training with the entire dataset when the labeled dataset reaches 85%.

**Figure 6:**
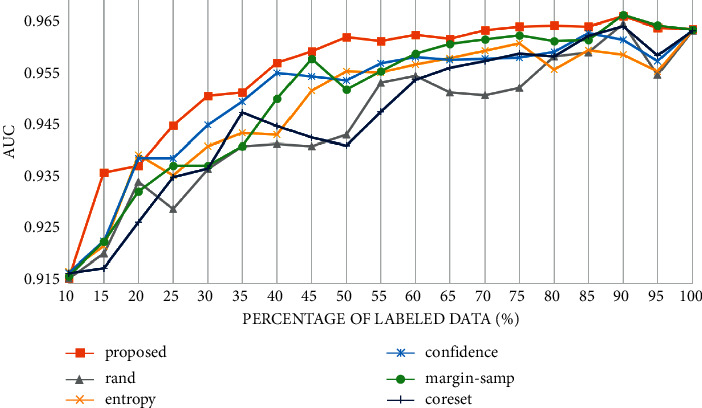
AUC curve of different selection strategies on PCam. To demonstrate the generality of the proposed framework, we also conduct experiments on multiclass datasets, including MNIST [38] and CIFAR10 [39].

To further compare the performance of the proposed method, we calculate the receiver operating characteristic curve (ROC) and area under the curve (AUC) of different active learning strategies after each iteration. The experimental results are shown in Figure 6. In Figure 6, the results of the proposed method and the uncertainty-based and diversity-based strategies are compared. The performance of the proposed strategy improves significantly in the first half of the iteration process. In the second half, with the increase in the sample size, the performance obtained by various methods gradually flattened. Even in the second half, the proposed strategy maintained a higher AUC.

**Figure 7:**
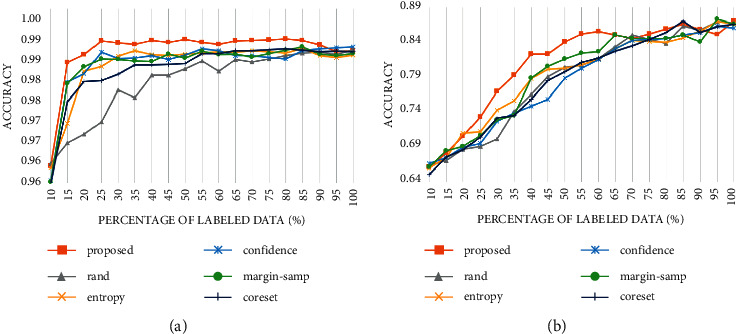
Accuracy curve of different selection strategies on (a) MNIST and (b) CIFAR10.

The result is shown in Figure 7.

The results on CIFAR10 and MNIST also support the effectiveness of our method. Although with the increase in the amount of data, various selection strategies gradually achieved close performance. However, in the early stage of iteration, the performance of the proposed strategy outperforms other strategies significantly, which shows its application value in reducing the cost of labeling. The proposed selection strategy is at most 2.04% higher than the random selection strategy on MNIST data (when the number of labeled samples accounts for 15%) and is higher than other strategies by up to 0.5% (when the labeled dataset accounts for 15%). On the CIFAR10 dataset, it is at most 6.77% higher than the random selection strategy (when the labeled dataset accounts for 30%) and 3.68% higher than other selection strategies (when the labeled dataset accounts for 30%).

To verify that the strategy that introduces the difference in the distribution of labeled data and unlabeled data is better than the pure hybrid strategy based on uncertainty and representative, we compare the selection efficiency of the proposed strategy and the hybrid strategy.

**Figure 8:**
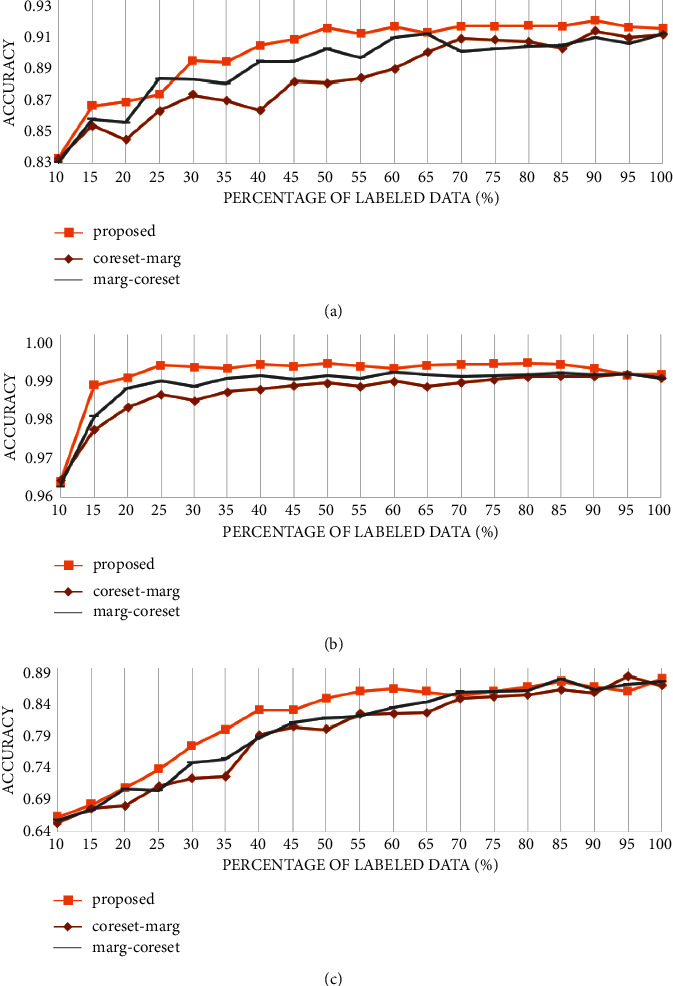
Comparison of proposed strategies and hybrid strategies in different datasets: (a) PCam, (b) MNIST, and (c) CIFAR10.

Assume that the number of samples queried in each iteration is *n* (here *n* is 5% of all samples). As shown in Figure 8, the “coreset-marg” strategy means the coreset method is used to select 2n samples from *𝒟*^*𝒰*_*i*_^ and then select *n* samples based on the uncertainty of margin sampling. The “marg-coreset” method first selects 2*n* samples from *𝒟*^*𝒰*_*i*_^ based on the uncertainty of margin sampling and then uses the coreset method to select *n* samples. Strategy that focuses on uncertainty first is better than that focuses on representative first. The strategy that combines the distribution differences in *𝒟*^*𝒰*_*i*_^ and *𝒟*^ℒ_*i*_^ performs better than the other two, which proves the validity of the introduction of the discriminator of *D*.

### 4.3. The Effectiveness of the Update Strategy

We compare the proposed update strategy with two other incremental update strategies. The first is to train the model with newly selected samples with the learning rate becoming one-fifth of the original, denoted as “queried only.” In the second strategy, in addition to using the newly selected data for training, it trains the task model with the old labeled samples whose model prediction and real label differ greatly. This error-based selection is denoted as “mistake replay.” The mistake replay strategy selects 40% old labeled samples in each iteration. The proposed update strategy freezes 70% of weight in the dropout layers while training in addition to keeping the learning rate decay to one-fifth of the original. The training time and accuracy of each iteration are recorded. The pros and cons of the strategy are measured through training time and accuracy drop. The experimental platform is a server with a 15-Core AMD EPYC 7543 32-Core Processor, 80 GB RAM, and an RTX 3090 GPU.

**Figure 9:**
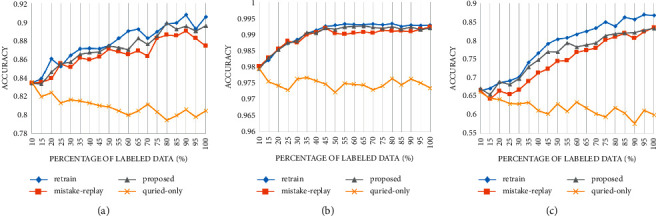
Accuracy of different update methods in different datasets under random selection strategy: (a) PCam, (b) MNIST, and (c) CIFAR10.

Figure 9 shows the accuracy change when querying by random selection strategy. The “retrain” series uses all the labeled data (old labeled and newly labeled) for training each time, which is the upper bound of other update strategies. Training with only the queried data does not improve the performance of the model but shows a slight downward trend. Both the mistake replay strategy and the proposed strategy can avoid the performance degradation caused by training only with query data. The accuracy under the proposed strategy is only slightly decreased compared with retraining with all data (Table 1).

**Figure 10:**
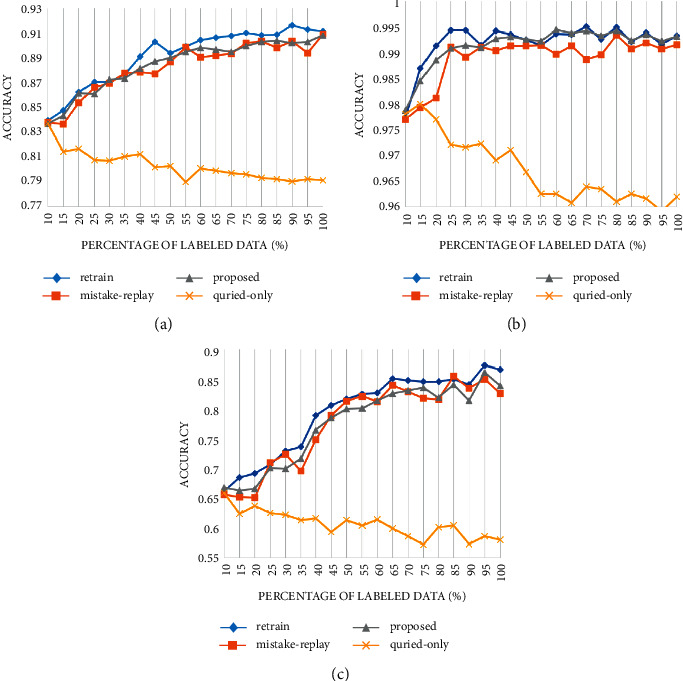
Accuracy of different update methods in different datasets under margin sampling selection strategy.

To further verify its effectiveness, we use the margin sampling-based strategy to carry out experiments, and the results are shown in Figure 10.

Similar results are obtained on the margin sampling strategy. The performance degradation caused by training only with query data was more prominent in the margin sampling strategy. This may be attributed to the distribution difference between the samples selected by margin sampling strategy and all data, while the random selection does not have such bias (Table 2).

Figures 9 and 10 show that under various datasets and query strategies, the proposed fine-tuning strategy achieves close performance with the mistake replay strategy, but our proposed method consumes a similar amount of time to update with only query samples. Its update cost is far from lower than retraining and mistake replay strategies.

## 5. Conclusion

The construction of an auxiliary medical image system requires a large amount of labeled data, which requires expensive annotation costs. In this study, based on the prediction of lymph node metastasis in breast cancer, an efficient active learning selection strategy is proposed. Its effectiveness is verified on other classification datasets. The three-stage selection strategy proposed in this study is an improvement on the traditional uncertainty-based selection. In particular, samples with large uncertainty are firstly selected according to the uncertainty measure, then the redundancy of the samples to be labeled is reduced by the coreset-based method, and finally, the discriminator of the distribution difference between the labeled samples and the unlabeled samples further filters the samples. This selection strategy, which takes into account the distribution differences between labeled samples and unlabeled samples, will try to eliminate such differences. Compared with simply using uncertainty strategies, representative strategies, or hybrid strategies, it has greater labeling efficiency. On the breast cancer lymph node dataset, only 50% of the data is used to achieve the effect of using all the data for training. Aiming at the problem that retraining consumes a lot of time in the model update process, we propose a dropout-based fine-tuning method, which achieves similar performance as the mistake replay update method but reduces training cost by an average of 79.87%. Compared with the retraining update strategy, training cost is reduced by 90.07% on average without causing excessive accuracy loss.

## Figures and Tables

**Algorithm 1:**
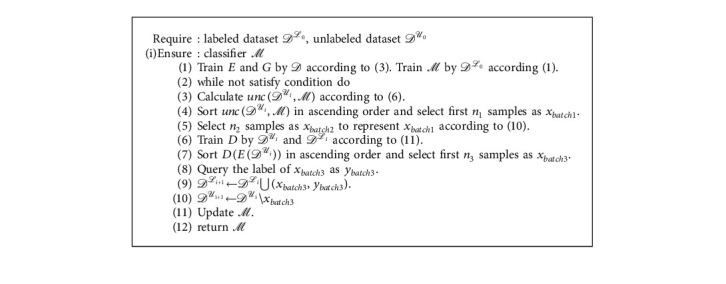
Procedure of the proposed framework.

**Table 1:** Time consumption (in seconds) of different update methods under random selection strategy.

	Retrain	Mistake replay	Proposed	Queried only
PCam	291.42	145.23	29.23	29.76
MNIST	387.17	192.93	38.73	36.63
CIFAR10	380.19	188.20	37.07	37.12

**Table 2:** Time consumption (in seconds) of different update methods under the margin sampling strategy.

	Retrain	Mistake replay	Proposed	Queried only
PCam	296.16	142.50	28.92	28.66
MNIST	387.96	190.98	38.67	38.99
CIFAR10	378.62	187.45	38.09	37.83

## Data Availability

The data used to support the findings of this study are currently under embargo, while the research findings are commercialized. Requests for data, 12 months after publication of this article, will be considered by the corresponding author.

## References

[1] Bejnordi B. E., Veta M., Van Diest P. J. (2017). Diagnostic assessment of deep learning algorithms for detection of lymph node metastases in women with breast cancer. *JAMA*.

[2] Yu G., Chen Z., Wu J., Tan Y. (Dec. 2021). Medical decision support system for cancer treatment in precision medicine in developing countries. *Expert Systems with Applications*.

[3] Yu G., Chen Z., Wu J., Tan Y. (2021). A diagnostic prediction framework on auxiliary medical system for breast cancer in developing countries. *Knowledge-Based Systems*.

[4] Yu G., Wu J. (Jan. 2022). Efficacy Prediction Based on Attribute and Multi-Source Data Collaborative for Auxiliary Medical System in Developing Countries. *Neural Computing and Applications*.

[5] Chang L., Wu J., Moustafa N., Bashir A. K., Yu K. (2021). AI-driven synthetic biology for non-small cell lung cancer drug effectiveness-cost analysis in intelligent assisted medical systems. *IEEE Journal of Biomedical and Health Informatics*.

[6] Cui R., Chen Z., Wu J., Tan Y., Yu G. (May 2021). A multiprocessing scheme for PET image pre-screening, noise reduction, segmentation and lesion partitioning. *IEEE Journal of Biomedical and Health Informatics*.

[7] Wu J., Tan Y., Chen Z., Zhao M. (2018). Data decision and drug therapy based on non-small cell lung cancer in a big data medical system in developing countries. *Symmetry*.

[8] Wu J., Chang L., Yu G. (Dec. 2021). Effective data decision-making and transmission system based on mobile health for chronic disease management in the elderly. *IEEE Systems Journal*.

[9] Wu J., Zhuang Q., Tan Y. (2020). Auxiliary medical decision system for prostate cancer based on ensemble method. *Computational and Mathematical Methods in Medicine*.

[10] Yang L., Zhang Y., Chen J., Zhang S., Chen D. Z. Suggestive annotation: a deep active learning framework for biomedical image segmentation.

[11] Wang J., Yan Y., Zhang Y., Cao G., Yang M., Ng M. K. Deep reinforcement active learning for medical image classification.

[12] Yuan P., Mobiny A., Jahanipour J., Li X., Cicalese P. A., Roysam B., Patel V. M., Dragan M., Nguyen H. V. Few is enough: task-augmented active meta-learning for brain cell classification.

[13] Ren P., Xiao Y., Chang X. (2022). A survey of deep active learning. *ACM Computing Surveys*.

[14] Zhou Z., Shin J., Zhang L., Gurudu S., Gotway M., Liang J. Fine-tuning convolutional neural networks for biomedical image analysis: actively and incrementally.

[15] van de Ven G. M., Tolias A. S. (2019). Three scenarios for continual learning. https://arxiv.org/abs/1904.07734.

[16] Jiao Y., Qi H. (2022). Capsule network assisted electrocardiogram classification model for smart healthcare. *Biocybernetics and Biomedical Engineering*.

[17] Smailagic A., Costa P., Gaudio A., Walawalkar D. (2020). O‐MedAL: Online active deep learning for medical image analysis. *Wiley Interdisciplinary Reviews: Data Mining and Knowledge Discovery*.

[18] Zhao Z., Yang X., Veeravalli B., Zeng Z. Deeply supervised active learning for finger bones segmentation.

[19] Zhou Z., Shin J. Y., Gurudu S. R., Gotway M. B., Liang J. (Jul. 2021). Active, continual fine tuning of convolutional neural networks for reducing annotation efforts. *Medical Image Analysis*.

[20] Li H., Yin Z. Attention, Suggestion and Annotation: A Deep Active Learning Framework for Biomedical Image Segmentation.

[21] Zhang Y., Yang L., Chen J., Fredericksen M., Hughes D. P., Chen D. Z. Deep adversarial networks for biomedical image segmentation utilizing unannotated images.

[22] Beluch W. H., Genewein T., Nurnberger A., Kohler J. M. The power of ensembles for active learning in image classification.

[23] Ranganathan H., Venkateswara H., Chakraborty S., Panchanathan S. Deep active learning for image classification.

[24] Yang W., Wu J., Luo J (2020). Effective data transmission and control based on social communication in social opportunistic complex networks. *Complexity*.

[25] Gal Y., Ghahramani Z. Dropout as a bayesian approximation: representing model uncertainty in deep learning.

[26] Gal Y., Islam R., Ghahramani Z. Deep bayesian active learning with image data.

[27] Wu J., Zhao M. (2020). An efficient data packet iteration and transmission algorithm in opportunistic social networks. *Journal of Ambient Intelligence and Humanized Computing*.

[28] Wu J., Tan Y., Tan Y. (June 2019). Hospital evaluation mechanism based on mobile health for IoT system in social networks. *Computers in Biology and Medicine*.

[29] Wu J., Chen Z., Zhao M. (2019). Information cache management and data transmission algorithm in opportunistic social networks. *Wireless Networks*.

[30] Wu J., Zhao M. (2019). Weight distribution and community reconstitution based on communities communications in social opportunistic networks. *Peer-to-Peer Networking and Applications*.

[31] Kirsch A., van Amersfoort J., Gal Y. (2019). BatchBALD: efficient and diverse batch acquisition for deep Bayesian active learning. *Advances in Neural Information Processing Systems*.

[32] Wang Z., Ye J. Querying discriminative and representative samples for batch mode Active Learning. *ACM Transactions on Knowledge Discovery from Data (TKDD)*.

[33] Ash J. T., Zhang C., Krishnamurthy A., Langford J., Agarwal A. (2019). Deep Batch Active Learning by Diverse, Uncertain Gradient Lower Bounds. https://arxiv.org/abs/1906.03671.

[34] Shui C., Zhou F., Gagné C., Wang B. Deep Active Learning: Unified and Principled Method for Query and Training.

[35] Kingma D. P., Welling M. Auto-encoding variational bayes.

[36] Zhan X., Long H., Gou F., Duan X., Kong G., Wu J. (2021). A convolutional neural network-based intelligent medical system with sensors for assistive diagnosis and decision-making in non-small cell lung cancer. *Sensors*.

[37] Veeling B. S., Linmans J., Winkens J., Cohen T., Welling M. Rotation equivariant CNNs for digital pathology.

[38] Lecun Y., Bottou L., Bengio Y., Haffner P. (1998). Gradient-based learning applied to document recognition. *Proceedings of the IEEE*.

[39] Krizhevsky A. (May 2012). *Learning Multiple Layers of Features from Tiny Images*.

